# Risk factors for disease generalization in acetylcholine receptor antibody-positive ocular myasthenia: a multicenter retrospective study

**DOI:** 10.3389/fneur.2026.1802425

**Published:** 2026-05-25

**Authors:** Lorenzo Verriello, Roberto Sartor, Fabrizio Bellizzi, Chiara Dalla Torre, Maria Elena Pessa, Marco Belluzzo, Alessio Bratina, Magda Quagliotto, Chiara Rosa Mancinelli, Michele Rana, Fulvio Pasquin, Martina Fabris, Giada Pauletto, Miriam Isola, Maria De Martino, Paolo Manganotti, Mariarosaria Valente

**Affiliations:** 1 Neurology Unit, Department of Head-Neck and Neurosciences, Santa Maria della Misericordia University Hospital, Udine, Italy; 2 Severe Acquired Brain Injury Unit, Department of Rehabilitation Medicine, Institute of Physical and Rehabilitation Medicine, University Hospital of Udine, Udine, Italy; 3 University of Udine, Udine, Italy; 4 Clinical Neurology Unit, Department of Head-Neck and Neurosciences, Santa Maria della Misericordia University Hospital, Udine, Italy; 5 Clinical Neurology Unit, Department of Medical, Surgical and Health Sciences, University of Trieste, Trieste, Italy; 6 Neurology Unit, Department of Medical, Gorizia-Monfalcone Hospital, Gorizia, Italy; 7 Department of Medicine (DMED), Institute of Clinical Pathology, University of Udine, Udine, Italy; 8 Division of Medical Statistic, Department of Medicine (DMED), University of Udine, Udine, Italy; 9 Department of Medicine (DMED), University of Udine, Udine, Italy

**Keywords:** anti-AChR antibodies, ocular myasthenia gravis, predictive factors, repetitive nerve stimulation, secondary generalization

## Abstract

**Objective:**

This study aimed to identify factors associated with secondary generalization in patients with ocular-onset myasthenia gravis (OoMG) who were positive for anti–acetylcholine receptor (AChR) antibodies. Early identification of patients at higher risk of progression to generalized myasthenia gravis (gMG) is clinically relevant to optimize therapeutic strategies and improve long-term outcomes.

**Methods:**

We conducted a multicenter retrospective cohort study including patients with anti-AChR–positive OoMG recruited from three Italian neurology centers. Demographic, clinical, electrophysiological, and serological data were collected. Univariable and multivariable Cox regression analyses were performed to identify factors associated with disease generalization.

**Results:**

Among 85 patients, 40 (47.1%) developed gMG during follow-up. In univariable Cox regression, higher anti-AChR antibody titers (HR 3.37, 95% CI 1.53–7.43; *p* = 0.002) and abnormal repetitive nerve stimulation of facial muscles (HR 2.49, 95% CI 1.16–5.34; *p* = 0.020) were significantly associated with secondary generalization. However, no independent predictors were identified in multivariable analysis.

**Conclusion:**

Higher anti-AChR antibody titers and abnormal facial repetitive nerve stimulation were associated with an increased risk of secondary generalization in seropositive OoMG. Although these factors did not independently predict generalization in multivariable analysis, their combined assessment may support early clinical risk stratification. Larger prospective studies are warranted to validate these findings and refine prognostic assessment.

## 1 Introduction

Myasthenia gravis (MG) is a chronic autoimmune neuromuscular disorder caused by impaired synaptic transmission at the neuromuscular junction. Its pathophysiology involves the production of autoantibodies against proteins located on the postsynaptic membrane. The autoantibodies associated with MG primarily include anti-acetylcholine receptor (AChR) antibodies, which are the most common (80–85% in generalized and 50–60% in ocular forms), anti-muscle-specific kinase (MuSK) antibodies (5–8%), and anti-lipoprotein-related protein 4 (LRP4) antibodies (1–5%). Anti-AChR antibodies mediate disease through three main pathogenic mechanisms: complement-mediated structural damage at the neuromuscular junction, internalization and degradation of AChRs, and functional blockade of the receptors (1). Thymic abnormalities are believed to play a central role in the loss of immunological tolerance leading to the production of anti-AChR antibodies (2). Approximately 50% of patients present with ocular symptoms as the initial manifestation of the disease, including ptosis and/or diplopia, a condition referred to as ocular-onset myasthenia gravis (OoMG). In some cases, the disease remains confined to ocular muscles (oMG), whereas in others it progresses to generalized myasthenia gravis (gMG) (3). The main therapeutic goals in OoMG patients are symptoms’ control and improvement of quality of life. Although acetylcholinesterase inhibitors and corticosteroids are widely used, the role of early immunosuppression and thymectomy in reducing the risk of secondary generalization remains controversial. Identifying reliable predictors of progression to gMG would therefore be clinically relevant, as it could support early risk stratification and guide therapeutic decision-making.

The aim of this multicenter study was to identify clinical and laboratory factors associated with secondary generalization in patients with anti-AChR antibody-positive OoMG.

## 2 Materials and methods

### 2.1 Study design

The study was conducted as a multicenter retrospective cohort investigation aimed at identifying clinical and laboratory factors associated with the development of gMG in patients with OoMG who were positive for anti-AChR antibodies. The study was approved by the local Ethics Committee (protocol IRB: 223/2025) and was conducted in accordance with the Declaration of Helsinki and the Good Clinical Practice guidelines.

### 2.2 Participants

Patients were identified through a retrospective review of clinical records from three centers: the Neurology Unit and the Clinical Neurology Unit of Udine University Hospital (Udine, Italy), the Clinical Neurology Unit of Trieste University Hospital (Trieste, Italy) and the Neurology Unit of Gorizia-Monfalcone Hospital (Gorizia, Italy). Inclusion criteria were: (1) confirmed diagnosis of MG based on the combination of clinical and laboratory findings, according to established guidelines (4); (2) ocular-onset of the disease; (3) positivity for anti-AChR antibodies; and (4) minimum follow-up duration of 2 years from disease onset in patients who had not developed gMG. Exclusion criteria included: positivity for anti-MUSK antibodies, seronegative MG, non-ocular disease onset, and congenital myasthenic syndromes.

### 2.3 Clinical and laboratory variables

Potential factors associated with secondary generalization were selected based on previous studies (5–11) and included: sex, age at onset, type of presenting ocular symptoms (unilateral or bilateral ptosis, diplopia, or both ptosis and diplopia), neurophysiological findings on 3 Hz repetitive nerve stimulation (RNS), anti-AChR antibodies titers, type of therapy at onset (acetylcholinesterase inhibitors alone or in combination with immunosuppressive treatment), presence of autoimmune comorbidities (either pre-existing or developed during follow-up), and thymic abnormalities.

Clinical data were obtained from neurological assessments and follow-up visits conducted between January 2005 and April 2025.

Based on age at onset, patients were classified as early-onset (<50 years) or late-onset (≥ 50 years).

Repetitive nerve stimulation (RNS) at 3 Hz was performed at symptom onset, prior to the initiation of any treatment, including pyridostigmine, corticosteroids, or other immunosuppressive therapies, as part of the diagnostic workup for MG. Recordings were obtained from the *orbicularis oculi* and/or *nasalis* muscles. The protocol included a post-exercise facilitation test, consisting of 20 s of voluntary contraction of the target muscle prior to stimulation. RNS positivity (RNS+) was defined as a decremental response ≥10% between the first and fourth stimuli at baseline or, when baseline findings were normal or borderline, after post-exercise testing (4). Baseline and post-exercise results were combined and analyzed as a single binary variable (RNS+).

Serum AChR antibody titers were measured at baseline in all patients, prior to the initiation of corticosteroids or other immunosuppressive therapies. All samples were analyzed in a centralized laboratory using the same standardized radioimmunoassay (RIA), ensuring analytical consistency across the participating centers. The assay was performed according to the manufacturer’s standard protocol (12). Titers >0.5 nMol/L were considered positive. Positive cases were further stratified into low-titer (0.5–5.0 nMol/L) and high-titer (>5.0 nMol/L) groups, based on the upper limit of the assay’s linear range. The cutoff was defined *a priori* to facilitate stratification of patients according to antibody burden and to improve interpretability of the analyses. This threshold also reflects the upper limit of the assay’s linear range, providing a technical rationale for its selection.

Thymic abnormalities were defined as the presence of thymic hyperplasia or thymoma, as detected by chest computed tomography or pathological confirmation after thymectomy.

Progression to generalized myasthenia gravis (gMG) was defined as the development of clinically evident weakness involving non-ocular muscle groups, including bulbar, limb, or respiratory muscles, corresponding to at least Myasthenia Gravis Foundation of America (MGFA) class II. Generalization was confirmed by a neurologist during clinical evaluation.

Time to generalization was defined as the interval between disease onset and the first clinical visit documenting generalized weakness. When available, patient-reported onset of non-ocular symptoms was also considered, but the date of generalization was based on clinical confirmation

### 2.4 Statistical analysis

Descriptive and inferential statistical analyses were performed to evaluate factors associated with secondary generalization in patients with OoMG. Continuous variables were summarized as mean ± standard deviation (SD), while categorical variables were expressed as frequencies and percentages. Time to secondary generalization and follow-up duration were reported as median and interquartile range (IQR).

Comparisons between continuous variables were performed using the Student’s *t*-test or the Mann–Whitney *U* test, as appropriate. Associations between secondary generalization (dependent variable) and potential clinical and laboratory factors were initially assessed using contingency tables with Pearson’s chi-square test or Fisher’s exact test.

Univariable Cox regression analyses were conducted to estimate the association between individual variables of interest and the time to secondary generalization. Hazard ratios (HRs), 95% confidence intervals (CIs), and *p*-values were calculated. Kaplan–Meier estimates were assessed to determine time to secondary generalization.

A multivariable Cox regression model was then constructed including variables that were significant in univariable analyses to assess their independent associations while accounting for potential confounding and collinearity.

All statistical analyses were performed using STATA version 19, and a two-tailed *p*-value <0.05 was considered statistically significant.

## 3 Results

**Figure 1:**
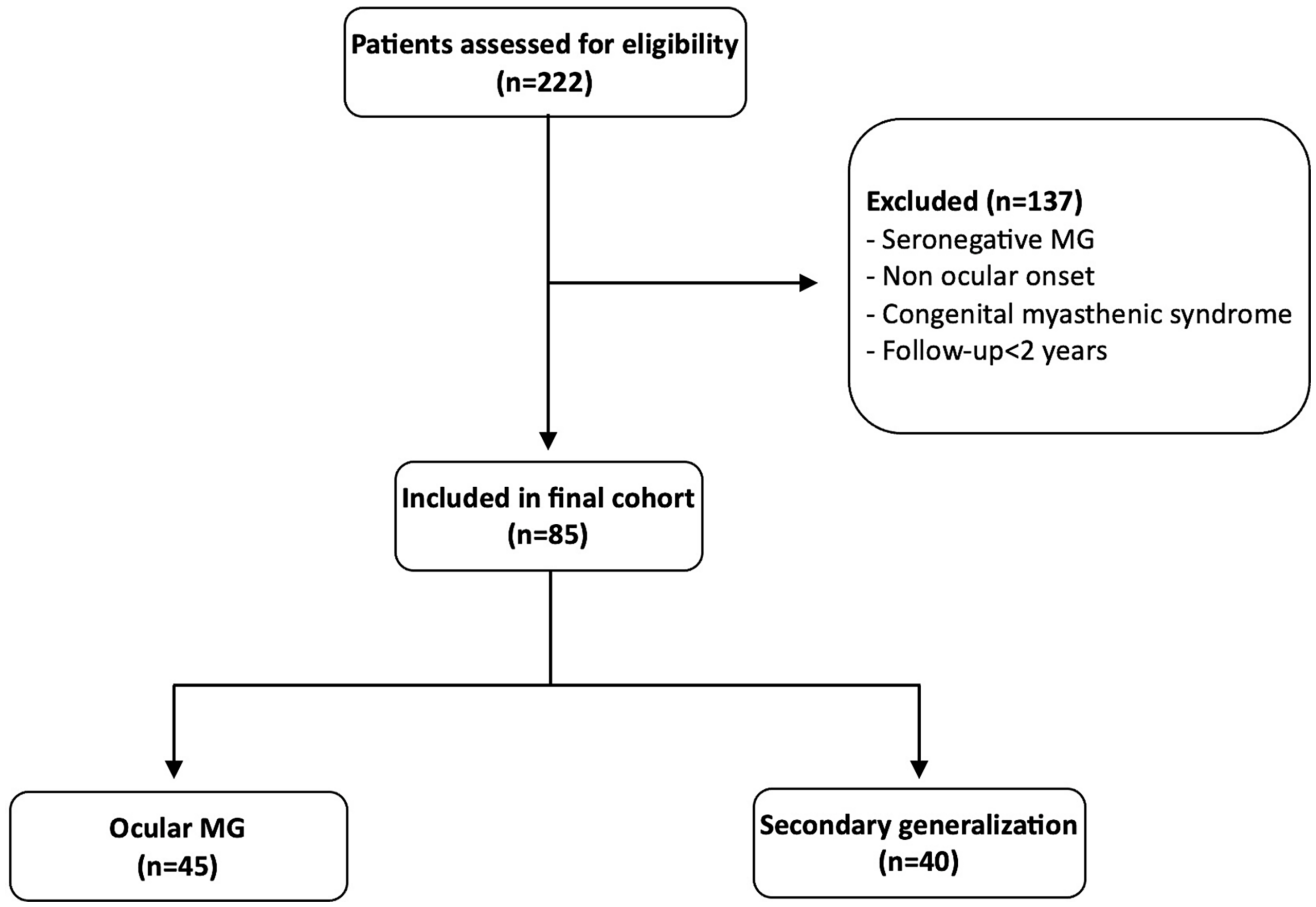
Patient selection flowchart.

A total of 222 patients were screened for eligibility in this multicenter study. Of these, 137 were excluded for not meeting inclusion criteria (seronegative MG, non-ocular onset, congenital myasthenic syndromes, or follow-up <2 years). The final cohort included 85 patients and consisted of 56 females and 29 males, with a mean age at disease onset of 64 years (SD 13.8). Median follow-up time was 38 months (IQR 11–78). During follow-up, 40 patients developed secondary generalization (47.1%), whereas 45 remained with purely ocular disease (Figure 1).

Baseline demographic, clinical, and instrumental characteristics of the study population are reported in Table 1, together with the unadjusted comparison between patients who remained purely ocular and those who developed secondary generalization. Among patients who experienced secondary generalization, the median time to conversion was 7 months (IQR 3–29 months), with 50% of events occurring within the first year and 67.5% within the first 24 months from disease onset.

**Table 1:** Demographic, clinical, and instrumental characteristics of the study cohort.

Demographic and clinical characteristics	Ocular MG	MG with secondary generalization	*p-*value	Available n
Sex, female/male	27/18	29/11	0.225	85
Age at onset (y), mean (SD)	63.2 (12.9)	65.0 (14.8)	0.563	85
Age at onset (y), mean (SD) stratified by sex				
Male	65.1 (12.6)	63.3 (11.8)	0.584	
Female	60.4 (13.1)	69.5 (21.3)	0.168	
Age at onset classification, n (%)			0.352	
Early onset (≤50 y)	9 (20%)	5 (12.5%)		
Late onset (>50 y)	36 (80%)	35 (87.5%)		
Presence of thymic diseases	4 (8.9%)	9 (22.5%)	0.082	85
Thymic hyperplasia	1 (2.2%)	4 (10%)		
Thymoma	3 (6.7%)	5 (12.5%)		
Other autoimmune disorders	17 (37.8%)	14 (35%)	0.791	85
Symptoms (baseline)			0.297	85
Only ptosis in one eye	10 (22.2%)	14 (35%)		
Only ptosis in both eyes	4 (8.9%)	1 (2.5%)		
Only diplopia	12 (26.7%)	12 (30%)		
Ptosis in one eye + diplopia	18 (40%)	10 (25%)		
Ptosis in both eyes + diplopia	1 (2.2%)	3 (7.5%)		
Neurophysiology, n (%)			0.014	69
RNS+	12 (26.7%)	18 (45%)		
RNS−	27 (60%)	12 (30%)		
RNS not performed	6 (13.3%)	10 (25%)		
First treatment, n (%)			0.396	85
No therapy	1 (2.2%)	4 (10%)		
Pyridostigmine	23 (51.1%)	16 (40%)		
Immunosuppressant	2 (4.4%)	1 (2.5%)		
Pyridostigmine + immunosuppressant	19 (42.2%)	19 (47.5%)		
High anti-AChR antibodies titers, n (%)	19 (42.2%)	32 (80%)	0.001	85

AChR, acetylcholine receptor; MG, myasthenia gravis; RNS, repetitive nerve stimulation.

Continuous variables are reported as mean (standard deviation) and categorical variables as number (percentage). Comparisons are performed between patients who remained ocular and those who developed secondary generalization. The “Available n” column indicates the number of patients with non-missing data for each variable. Missing data occurred only for repetitive nerve stimulation (RNS), which was unavailable in 16 patients due to incomplete electrophysiological assessment at disease onset. Age at onset was analyzed both as a continuous and categorical variable (early onset ≤50 years; late onset >50 years).

**Figure 2:**
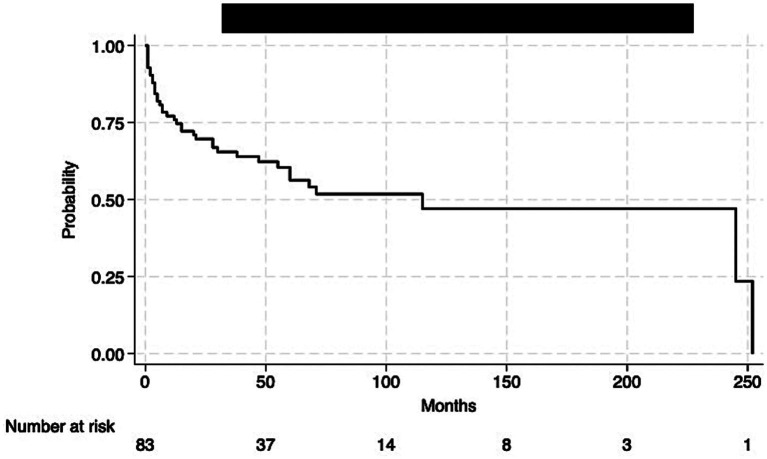
Kaplan–Meier curve of time to secondary generalization.

In the other group, the median follow-up time was 58 months (IQR 38–98). Considering the overall cohort, the cumulative probability of remaining free from secondary generalization was 75.8% at 1 year (95% CI, 65.2–83.7%) and 69.7% at 2 years (95% CI, 58.5–78.4%) (Figure 2).

### 3.1 Descriptive analysis and group comparison

No significant differences were observed between patients with and without secondary generalization with respect to sex distribution (*p* = 0.225) or age at disease onset. Specifically, age at onset did not differ when analyzed as a continuous variable (*p* = 0.563), nor when categorized as early- (<50 years) versus late-onset (≥50 years) (*p* = 0.352). Similarly, coexisting autoimmune diseases, type of ocular symptoms at onset, and initial therapeutic approach did not significantly differ between groups (Table 1).

Among the 45 patients who remained ocular during follow-up, four underwent thymectomy due to suspected thymic abnormalities on chest CT. In all cases, surgery was performed at disease onset. Histological examination revealed thymoma in three patients and thymic hyperplasia in one. Among the 40 patients who progressed to generalized myasthenia gravis, nine underwent thymectomy. In eight of these cases, surgery was performed after generalization had already occurred. Histological findings showed thymoma in five patients and thymic hyperplasia in four. Overall, thymic histology was available in a limited subset of patients. Thymic hyperplasia was observed in 4/40 (10%) patients who generalized and in 1/45 (2.2%) patients who remained ocular. Thymoma was found in 5/40 (12.5%) and 3/45 (6.7%) patients, respectively. Given the small number of patients with available histological data, these findings are reported descriptively. All patients who underwent thymectomy experienced disease relapses during follow-up.

Precise quantitative AChR titers were available in 34 patients, whereas in the remaining cases values were reported as >5 nMol/L. Consistent with our stratification approach, secondary generalization was more frequent among patients with titers >5 nMol/L compared to those with lower levels (62.7% vs. 23.5%, *p* < 0.05). Furthermore, within the low-titer group, exploratory analyses based on the median value (2.15 nMol/L) showed a gradient in the frequency of generalization, increasing from 17.6% in patients with titers <2.15 nMol/L to 29.4% in those with titers between 2.15 and 5 nMol/L.

Accordingly, elevated anti-AChR antibody titers (>5.0 nMol/L) were significantly more frequent among patients who developed secondary generalization compared with those who remained purely ocular (80% vs. 42.2%, *p* = 0.001). Likewise, abnormal RNS findings were more commonly observed in generalized patients (45%) than in non-generalized ones (26.7%, *p* = 0.014).

RNS data were unavailable in 16 patients, due to incomplete electrophysiological assessment at disease onset. Baseline demographic and clinical characteristics did not differ significantly between patients with available and unavailable RNS data (Supplementary Table S1).

### 3.2 Cox regression analyses

Results of univariable and multivariable Cox regression analyses, assessing factors associated with secondary generalization, are summarized in Table 2.

**Table 2:** Univariable and multivariable Cox regression analyses of factors associated with secondary generalization.

Variable	Univariable HR (95% CI)	*p* value	Multivariable HR (95% CI)	*p* value
High anti-AChR antibody titer (>5.0 nMol/L)	3.37 (1.53 – 7.43)	0.002	1.03 (0.51 – 2.07)	0.932
Abnormal RNS (>10% decrement)	2.49 (1.16 – 5.34)	0.020	0.86 (0.40 – 1.86)	0.713

AChR, acetylcholine receptor; CI, confidence interval; OR, odds ratio; RNS, repetitive nerve stimulation.

In univariable Cox regression analyses, both elevated anti-AChR antibody titers (HR 3.37, 95% CI 1.53–7.43; *p* = 0.002) and abnormal RNS findings (HR 2.49, 95% CI 1.16–5.34; *p* = 0.020) were significantly associated with an increased risk of secondary generalization.

When both variables were included in a multivariable logistic regression model, anti-AChR antibody titers (HR 1.03; *p* = 0.932) and abnormal RNS findings (HR 0.87; *p* = 0.713) did not reach independent statistical significance.

## 4 Discussion

In this multicenter retrospective cohort, higher anti-AChR antibody titers were strongly associated with an increased risk of secondary generalization. Previous studies have consistently shown that anti-AChR antibody positivity represents a major risk factor for disease generalization (8, 13–17). Other investigations showed that elevated anti-AChR titers are associated with a higher likelihood of progression from ocular to generalized disease (18, 19). In the largest cohort reported to date, Peeler et al. showed that patients who developed gMG exhibited significantly higher antibody levels and identified high titers as an independent risk factor for progression (20). Our findings are in line with this body of evidence and further support the clinical relevance of quantitative anti-AChR antibody assessment for risk stratification in OoMG.

The observed association between higher anti-AChR titers and disease generalization is consistent with the immunogenetic model proposed by Zhong et al. (21), suggesting that the magnitude of the autoimmune response, reflected by antibody titers, plays a central role in determining disease evolution.

This interpretation is supported by immunogenetic studies indicating that distinct HLA backgrounds influence both antibody production and clinical phenotype in MG (22, 23). Future studies integrating high-resolution HLA typing with serological and clinical data may further refine prognostic stratification and support the development of personalized management strategies.

In addition to antibody titers, abnormal decremental response on RNS of the facial muscles were associated with secondary generalization in our cohort. This finding is consistent with the observation reported by Ding et al. (24), but differs from those of Kim et al. (25), who identified abnormal RNS in limb muscles rather than facial muscles as a predictor of generalization. Several factors may account for this discrepancy. One possible explanation is that our cohort was restricted to anti-AChR-positive patients, whereas approximately one-third of the population analyzed by Kim et al. was seronegative. Given that anti-AChR antibody positivity is itself a recognized risk factor for secondary generalization, the more homogeneous serological profile of our cohort may have enhanced the prognostic value of facial muscle testing. Furthermore, facial muscle RNS abnormalities, even if not directly assessing extraocular muscles involvement, may reflect early subclinical neuromuscular junction dysfunction extending beyond purely ocular structures in seropositive patients, thus anticipating systemic disease extension. The homogeneity of our study population may therefore have facilitated the detection of associations that were less evident in more heterogeneous cohorts (25). Overall, these findings suggest that both facial and limb RNS abnormalities may provide prognostic information, although their relative contribution likely varies according to patients’ characteristics. Standardized prospective studies incorporating RNS assessment of multiple muscle groups are warranted to clarify this issue.

When anti-AChR antibody titers and RNS abnormalities were included simultaneously in multivariable analysis, they did not reach independent statistical significance. This attenuation may reflect sample size, missing neurophysiological data in some subjects, potential collinearity between predictors, and/or shared underlying pathophysiological mechanism. Although anti-AChR antibody titers and facial RNS abnormalities did not retain independent significance in multivariable analyses, their consistent association with secondary generalization in univariable models suggests a possible role in clinical risk assessment. However, any potential additive or synergistic effect should be interpreted with caution and regarded as hypothesis-generating. Further studies in larger cohorts are needed to clarify whether their combined evaluation may improve early identification of patients at higher risk of generalization.

Other variables commonly proposed as predictors - including age at onset, sex, autoimmune comorbidities, symptoms profile at presentation, and initial therapeutic approach—were not associated with secondary generalization in our cohort.

Thymic histology was available only in a limited subset of patients and was therefore analyzed descriptively without formal statistical comparisons. Thymoma and thymic hyperplasia were considered separately due to their distinct clinical relevance. However, the small sample size and the heterogeneity in the timing of thymectomy—often performed after disease generalization—limit the interpretation of these findings and introduce potential selection bias. Consequently, no conclusions can be drawn regarding the association between thymic pathology and disease progression in our cohort.

Our findings differ from those of prior studies (7–9, 14–17, 26) and may be explained by methodological differences, particularly the exclusive inclusion of anti-AChR-positive patients, which may reduce confounding factors and increase the specificity of observed associations.

### 4.1 Strengths and limitations

The main strengths of this study include the well-defined and homogeneous cohort restricted to anti-AChR-positive OoMG patients, which reduced clinical and immunological heterogeneity and enhanced internal validity. However, this approach limits the generalizability of our findings to other MG subgroups. The use of standardized diagnostic criteria and objective laboratory and neurophysiological measures further strengthened data reliability.

Regarding study’s limitations, the retrospective design may introduce selection and information bias, and the relatively small sample size limits generalizability and statistical power, particularly in multivariable analyses. In addition, the long enrollment period represents a potential limitation, as diagnostic and therapeutic approaches to MG have evolved over time. Nevertheless, all patients were evaluated and treated in specialized neuromuscular centers, according to standardized criteria, which may have mitigated the impact of these temporal changes. The lack of detailed longitudinal treatment data may have affected our ability to fully assess the relationship between immunosuppression and disease progression. Thymectomy was not analyzed as an independent variable, and its potential role as a confounding factor influencing disease progression cannot be excluded, particularly given the variability in surgical timing relative to disease onset. Finally, the lack of precise quantitative AChR measurements above the linear range of the RIA method did not allow identification of a definitive cutoff for generalization. However, the observed gradient in clinical outcomes across increasing antibody levels supports an association between antibody burden and disease severity. In this context, the >5 nMol/L threshold appears to represent a pragmatic cutoff for identifying patients at higher risk, although further studies with fully quantitative measurements are warranted.

## 5 Conclusion

Higher anti-AChR antibody titers and abnormal facial RNS findings were associated with an increased risk of secondary generalization in our cohort of patients with seropositive OoMG. Although these factors did not independently predict generalization in multivariable analysis, their combined assessment may support early clinical risk stratification. Larger prospective studies are needed to validate these findings and develop reliable prognostic tools for guiding personalized management strategies.

## Data Availability

The raw data supporting the conclusions of this article will be made available by the authors, without undue reservation.
