# Supplementary material for: Risk factors for disease generalization in acetylcholine receptor antibody-positive ocular myasthenia: a multicenter retrospective study
Source: Front Neurol. 2026 May 25;17:1802425. doi: 10.3389/fneur.2026.1802425 (PMC13243071; doi:10.3389/fneur.2026.1802425)
Supplement: Supplementary file 1 [file Table_1.DOCX]

**Supplementary Table S1. Baseline characteristics of patients with vs without available RNS data**

| **Variable** | **RNS available (n=69)** | **RNS not available (n=16)** | **p-value** |
| --- | --- | --- | --- |
| Age at onset, mean (SD) | 63.3 | 67.1 | 0.38 |
| Female sex, n (%) | 26 (37.7%) | 3 (18.8%) | 0.25 |
| Thymoma/thymic hyperplasia, n (%) | 11 (15.9%) | 2 (12.5%) | 0.99 |
| Ptosis at onset, n (%) | 51 (73.9%) | 11 (68.8%) | 0.91 |
| Diplopia at onset, n (%) | 46 (66.7%) | 10 (62.5%) | 0.98 |
| Low AChR antibody titer, n (%) | 28 (40.6%) | 6 (37.5%) | 0.80 |
| High AChR antibody titer, n (%) | 41 (59.4%) | 10 (62.5%) | 0.80 |
| Ocular myasthenia, n (%) | 39 (56.5%) | 6 (37.5%) | 0.18 |
| Generalization during follow-up, n (%) | 30 (43.5%) | 10 (62.5%) | 0.17 |
| Time to generalization (months), median (IQR) | 7.0 | 9.5 | 0.64 |

Abbreviations: RNS, repetitive nerve stimulation; AChR, acetylcholine receptor
